# Social Media Use and Its Connection to Mental Health: A Systematic Review

**DOI:** 10.7759/cureus.8627

**Published:** 2020-06-15

**Authors:** Fazida Karim, Azeezat A Oyewande, Lamis F Abdalla, Reem Chaudhry Ehsanullah, Safeera Khan

**Affiliations:** 1 Psychology, California Institute of Behavioral Neurosciences and Psychology, Fairfield, USA; 2 Business & Management, University Sultan Zainal Abidin, Terengganu, MYS; 3 Family Medicine, California Institute of Behavioral Neurosciences and Psychology, Fairfield, USA; 4 Family Medicine, Lagos State Health Service Commission/Alimosho General Hospital, Lagos, NGA; 5 Internal Medicine, California Institute of Behavioral Neurosciences and Psychology, Fairfield, USA

**Keywords:** social media, mental health, systematic review, prisma

## Abstract

Social media are responsible for aggravating mental health problems. This systematic study summarizes the effects of social network usage on mental health. Fifty papers were shortlisted from google scholar databases, and after the application of various inclusion and exclusion criteria, 16 papers were chosen and all papers were evaluated for quality. Eight papers were cross-sectional studies, three were longitudinal studies, two were qualitative studies, and others were systematic reviews. Findings were classified into two outcomes of mental health: anxiety and depression. Social media activity such as time spent to have a positive effect on the mental health domain. However, due to the cross-sectional design and methodological limitations of sampling, there are considerable differences. The structure of social media influences on mental health needs to be further analyzed through qualitative research and vertical cohort studies.

## Introduction and background

Human beings are social creatures that require the companionship of others to make progress in life. Thus, being socially connected with other people can relieve stress, anxiety, and sadness, but lack of social connection can pose serious risks to mental health [1].

Social media

**Figure 1:**
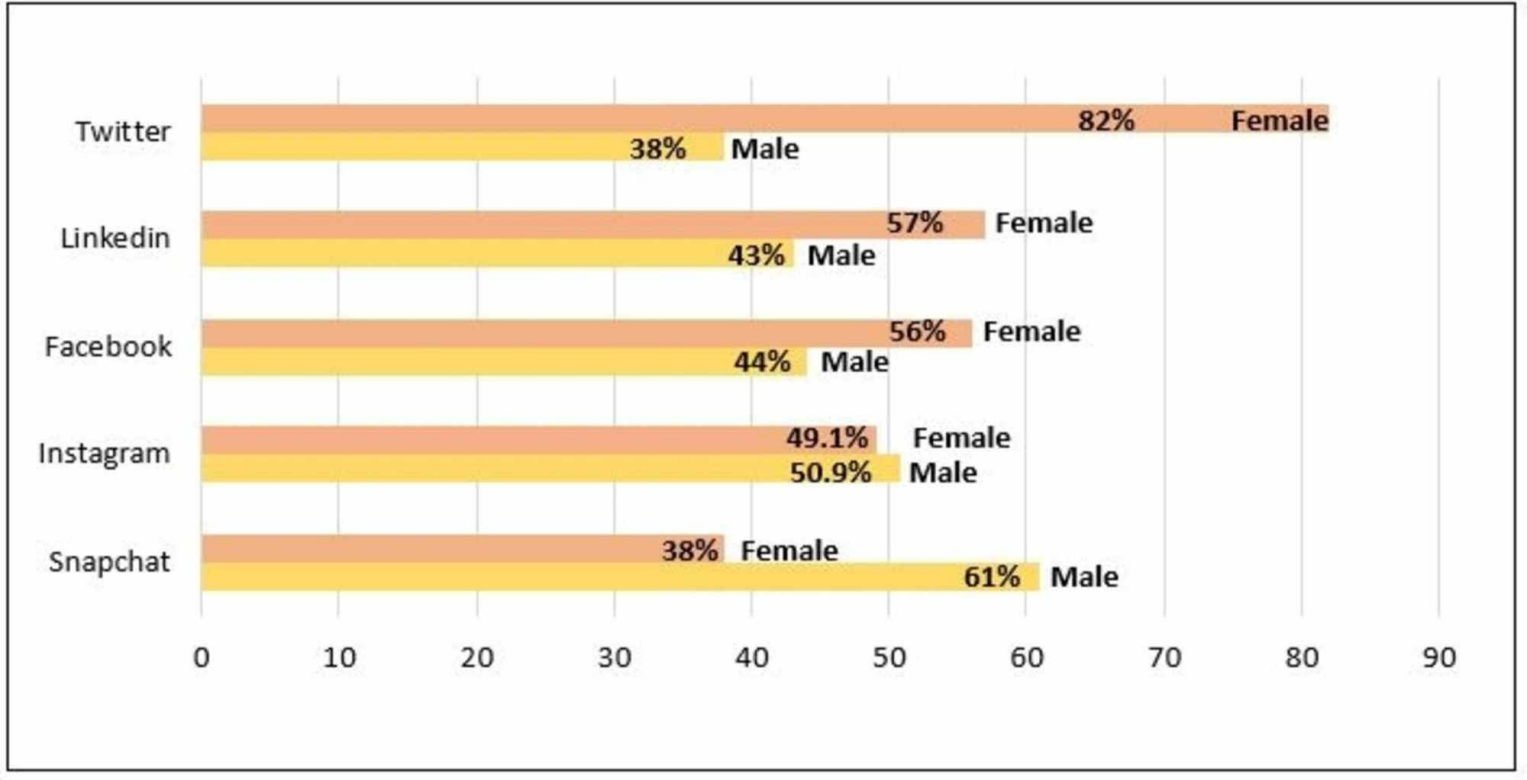
Gender distribution of social media audiences worldwide as of January 2020, sorted by platform

Social media has recently become part of people's daily activities; many of them spend hours each day on Messenger, Instagram, Facebook, and other popular social media. Thus, many researchers and scholars study the impact of social media and applications on various aspects of people’s lives [2]. Moreover, the number of social media users worldwide in 2019 is 3.484 billion, up 9% year-on-year [3-5]. A statistic in Figure 1 shows the gender distribution of social media audiences worldwide as of January 2020, sorted by platform. It was found that only 38% of Twitter users were male but 61% were using Snapchat. In contrast, females were more likely to use LinkedIn and Facebook. There is no denying that social media has now become an important part of many people's lives. Social media has many positive and enjoyable benefits, but it can also lead to mental health problems. Previous research found that age did not have an effect but gender did; females were much more likely to experience mental health than males [6,7].

Impact on mental health

Mental health is defined as a state of well-being in which people understand their abilities, solve everyday life problems, work well, and make a significant contribution to the lives of their communities [8]. There is debated presently going on regarding the benefits and negative impacts of social media on mental health [9,10]. Social networking is a crucial element in protecting our mental health. Both the quantity and quality of social relationships affect mental health, health behavior, physical health, and mortality risk [9]. The Displaced Behavior Theory may help explain why social media shows a connection with mental health. According to the theory, people who spend more time in sedentary behaviors such as social media use have less time for face-to-face social interaction, both of which have been proven to be protective against mental disorders [11,12]. On the other hand, social theories found how social media use affects mental health by influencing how people view, maintain, and interact with their social network [13]. A number of studies have been conducted on the impacts of social media, and it has been indicated that the prolonged use of social media platforms such as Facebook may be related to negative signs and symptoms of depression, anxiety, and stress [10-15]. Furthermore, social media can create a lot of pressure to create the stereotype that others want to see and also being as popular as others.

The need for a systematic review

Systematic studies can quantitatively and qualitatively identify, aggregate, and evaluate all accessible data to generate a warm and accurate response to the research questions involved [4]. In addition, many existing systematic studies related to mental health studies have been conducted worldwide. However, only a limited number of studies are integrated with social media and conducted in the context of social science because the available literature heavily focused on medical science [6]. Because social media is a relatively new phenomenon, the potential links between their use and mental health have not been widely investigated.

This paper attempt to systematically review all the relevant literature with the aim of filling the gap by examining social media impact on mental health, which is sedentary behavior, which, if in excess, raises the risk of health problems [7,9,12]. This study is important because it provides information on the extent of the focus of peer review literature, which can assist the researchers in delivering a prospect with the aim of understanding the future attention related to climate change strategies that require scholarly attention. This study is very useful because it provides information on the extent to which peer review literature can assist researchers in presenting prospects with a view to understanding future concerns related to mental health strategies that require scientific attention. The development of the current systematic review is based on the main research question: how does social media affect mental health?

## Review

Research strategy

The research was conducted to identify studies analyzing the role of social media on mental health. Google Scholar was used as our main database to find the relevant articles. Keywords that were used for the search were: (1) “social media”, (2) “mental health”, (3) “social media” AND “mental health”, (4) “social networking” AND “mental health”, and (5) “social networking” OR “social media” AND “mental health” (Table 1).

**Table 1:** Number of research articles for the searched keyword

Keyword/Combination of Keyword	Database	Number of Results
“social media”	Google Scholar	877,000
“mental health”	Google Scholar	633,000
“social media” AND “mental health”	Google Scholar	78,000
“social networking” AND “mental health”	Google Scholar	18,600
"social networking "OR "social media" AND "mental health"	Google Scholar	17,000

**Figure 2:**
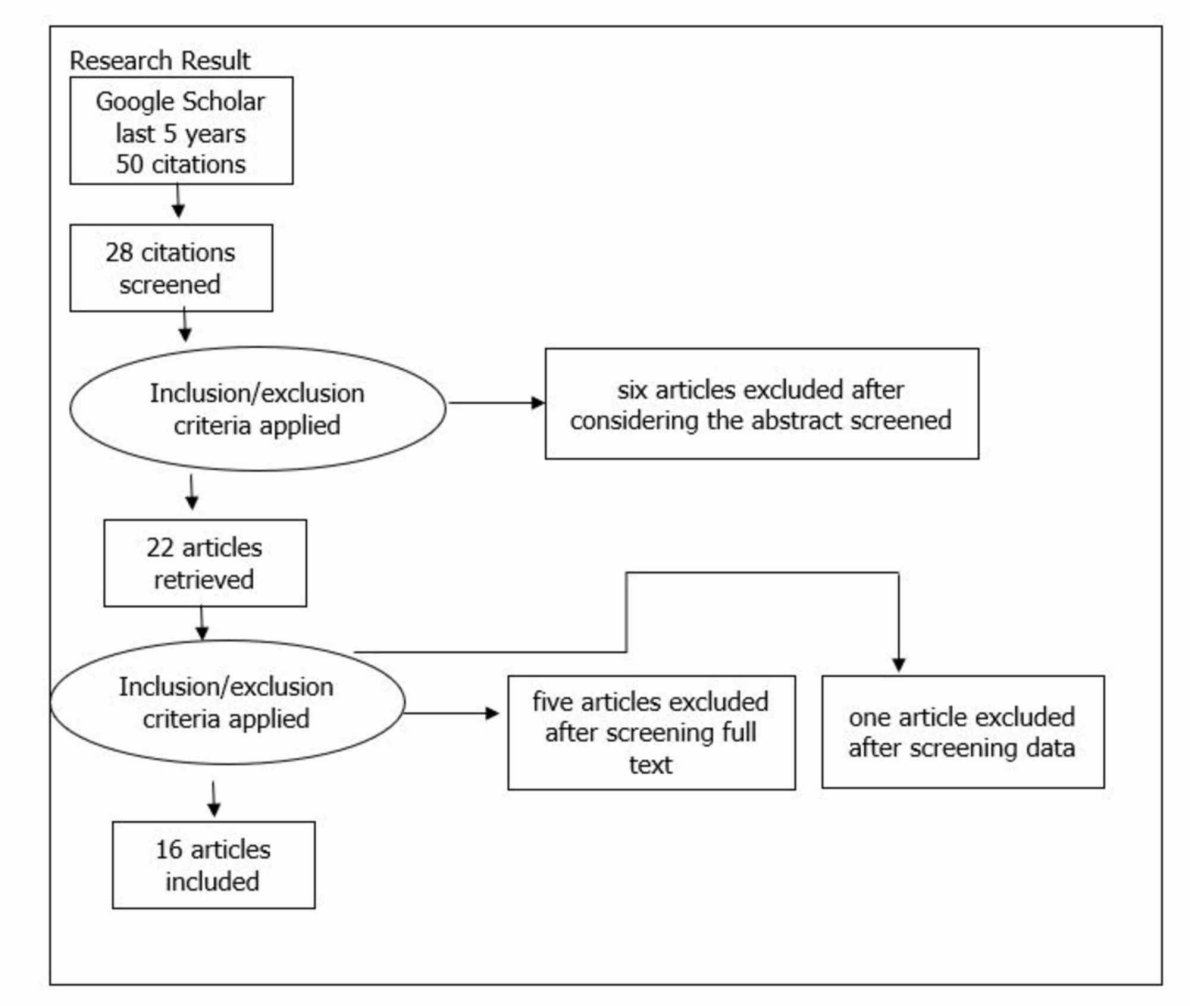
PRISMA diagram detailing the study identification and selection process PRISMA, Preferred Reporting Items for Systematic Reviews and Meta-Analyses

Out of the results in Table 1, a total of 50 articles relevant to the research question were selected. After applying the inclusion and exclusion criteria, duplicate papers were removed, and, finally, a total of 28 articles were selected for review (Figure 2).

Inclusion and exclusion criteria

Peer-reviewed, full-text research papers from the past five years were included in the review. All selected articles were in English language and any non-peer-reviewed and duplicate papers were excluded from finally selected articles.

Result

Of the 16 selected research papers, there were a research focus on adults, gender, and preadolescents [10-19]. In the design, there were qualitative and quantitative studies [15,16]. There were three systematic reviews and one thematic analysis that explored the better or worse of using social media among adolescents [20-23]. In addition, eight were cross-sectional studies and only three were longitudinal studies [24-29].The meta-analyses included studies published beyond the last five years in this population. Table 2 presents a selection of studies from the review.

**Table 2:** Some of the studies/papers included in the review IGU, internet gaming disorder; PSMU, problematic social media use

Author	Title of Study	Method	Findings
Berryman et al. [10]	Social Media Use and Mental Health among Young Adults	Cross-sectional	Social media use was not predictive of impaired mental health functioning.
Coyne et al. [11]	Does Time Spent using Social Media Impact Mental Health?: An Eight Year Longitudinal Study	8-year longitudinal study	Increased time spent on social media was not associated with increased mental health issues across development when examined at the individual level.
Escobar-Viera et al. [12]	For Better or for Worse? A Systematic Review of the Evidence on Social Media Use and Depression Among Lesbian, Gay, and Bisexual Minorities	Systematic Literature Review	Social media provides a space to disclose minority experiences and share ways to cope and get support; constant surveillance of one's social media profile can become a stressor, potentially leading to depression.
O’Reilly et al. [15]	Potential of Social Media in Promoting Mental Health in Adolescents	qualitative study	Adolescents frequently utilize social media and the internet to seek information about mental health.
O’Reilly [16]	Social Media and Adolescent Mental Health: The Good, the Bad and the Ugly	focus groups	Much of the negative rhetoric of social media was repeated by mental health practitioners, although there was some acknowledgement of potential benefit.
Feder et al. [17]	Is There an Association Between Social Media Use and Mental Health? The Timing of Confounding Measurement Matters	longitudinal	Frequent social media use report greater symptoms of psychopathology.
Rasmussen et al. [19]	The Serially Mediated Relationship between Emerging Adults’ Social Media Use and Mental Well-Being	Exploratory study	Social media use may be a risk factor for mental health struggles among emerging adults and that social media use may be an activity which emerging adults resort to when dealing with difficult emotions.
Keles et al. [19]	A Systematic Review: The Influence of Social Media on Depression, Anxiety and Psychological Distress in Adolescents	systematic review	Four domains of social media: time spent, activity, investment, and addiction. All domains correlated with depression, anxiety and psychological distress.
Nereim et al. [21]	Social Media and Adolescent Mental Health: Who You Are and What You do Matter	Exploratory	Passive social media use (reading posts) is more strongly associated with depression than active use (making posts).
Mehmet et al. [22]	Using Digital and Social Media for Health Promotion: A Social Marketing Approach for Addressing Co‐morbid Physical and Mental Health	Intervention	Social marketing digital media strategy as a health promotion methodology. The paper has provided a framework for implementing and evaluating the effectiveness of digital social media campaigns that can help consumers, carers, clinicians, and service planners address the challenges of rural health service delivery and the tyranny of distance,
Odgers and Jensen [23]	Adolescent Mental Health in the Digital Age: Facts, Fears, and Future Directions	Review	The review highlights that most research to date has been correlational, has focused on adults versus adolescents, and has generated a mix of often conflicting small positive, negative, and null associations.
Twenge and Martin [24]	Gender Differences in Associations between Digital Media Use and Psychological Well-Being: Evidence from Three Large Datasets	Cross-sectional	Females were found to be addicted to social media as compared with males.
Fardouly et al. [25]	The Use of Social Media by Australian Preadolescents and its Links with Mental Health	Cross-sectional	Users of YouTube, Instagram, and Snapchat reported more body image concerns and eating pathology than non-users, but did not differ on depressive symptoms or social anxiety
Wartberg et al. [26]	Internet Gaming Disorder and Problematic Social Media Use in a Representative Sample of German Adolescents: Prevalence Estimates, Comorbid Depressive Symptoms, and Related Psychosocial Aspects	Cross-sectional	Bivariate logistic regression analyses showed that more depressive symptoms, lower interpersonal trust, and family functioning were statistically significantly associated with both IGD and PSMU.
Neira and Barber [28]	Social Networking Site Use: Linked to Adolescents’ Social Self-Concept, Self-Esteem, and Depressed Mood	Cross-sectional	Higher investment in social media (e.g. active social media use) predicted adolescents’ depressive symptoms. No relationship was found between the frequency of social media use and depressed mood.

Discussion

This study has attempted to systematically analyze the existing literature on the effect of social media use on mental health. Although the results of the study were not completely consistent, this review found a general association between social media use and mental health issues. Although there is positive evidence for a link between social media and mental health, the opposite has been reported.

For example, a previous study found no relationship between the amount of time spent on social media and depression or between social media-related activities, such as the number of online friends and the number of “selfies”, and depression [29]. Similarly, Neira and Barber found that while higher investment in social media (e.g. active social media use) predicted adolescents’ depressive symptoms, no relationship was found between the frequency of social media use and depressed mood [28].

In the 16 studies, anxiety and depression were the most commonly measured outcome. The prominent risk factors for anxiety and depression emerging from this study comprised time spent, activity, and addiction to social media. In today's world, anxiety is one of the basic mental health problems. People liked and commented on their uploaded photos and videos. In today's age, everyone is immune to the social media context. Some teens experience anxiety from social media related to fear of loss, which causes teens to try to respond and check all their friends' messages and messages on a regular basis.

On the contrary, depression is one of the unintended significances of unnecessary use of social media. In detail, depression is limited not only to Facebooks but also to other social networking sites, which causes psychological problems. A new study found that individuals who are involved in social media, games, texts, mobile phones, etc. are more likely to experience depression.

The previous study found a 70% increase in self-reported depressive symptoms among the group using social media. The other social media influence that causes depression is sexual fun [12]. The intimacy fun happens when social media promotes putting on a facade that highlights the fun and excitement but does not tell us much about where we are struggling in our daily lives at a deeper level [28]. Another study revealed that depression and time spent on Facebook by adolescents are positively correlated [22]. More importantly, symptoms of major depression have been found among the individuals who spent most of their time in online activities and performing image management on social networking sites [14].

Another study assessed gender differences in associations between social media use and mental health. Females were found to be more addicted to social media as compared with males [26]. Passive activity in social media use such as reading posts is more strongly associated with depression than doing active use like making posts [23]. Other important findings of this review suggest that other factors such as interpersonal trust and family functioning may have a greater influence on the symptoms of depression than the frequency of social media use [28,29].

Limitation and suggestion

The limitations and suggestions were identified by the evidence involved in the study and review process. Previously, 7 of the 16 studies were cross-sectional and slightly failed to determine the causal relationship between the variables of interest. Given the evidence from cross-sectional studies, it is not possible to conclude that the use of social networks causes mental health problems. Only three longitudinal studies examined the causal relationship between social media and mental health, which is hard to examine if the mental health problem appeared more pronounced in those who use social media more compared with those who use it less or do not use at all [19,20,24]. Next, despite the fact that the proposed relationship between social media and mental health is complex, a few studies investigated mediating factors that may contribute or exacerbate this relationship. Further investigations are required to clarify the underlying factors that help examine why social media has a negative impact on some peoples’ mental health, whereas it has no or positive effect on others’ mental health.

## Conclusions

Social media is a new study that is rapidly growing and gaining popularity. Thus, there are many unexplored and unexpected constructive answers associated with it. Lately, studies have found that using social media platforms can have a detrimental effect on the psychological health of its users. However, the extent to which the use of social media impacts the public is yet to be determined. This systematic review has found that social media envy can affect the level of anxiety and depression in individuals. In addition, other potential causes of anxiety and depression have been identified, which require further exploration.

The importance of such findings is to facilitate further research on social media and mental health. In addition, the information obtained from this study can be helpful not only to medical professionals but also to social science research. The findings of this study suggest that potential causal factors from social media can be considered when cooperating with patients who have been diagnosed with anxiety or depression. Also, if the results from this study were used to explore more relationships with another construct, this could potentially enhance the findings to reduce anxiety and depression rates and prevent suicide rates from occurring.
